# NF-κB-Induced IL-6 Ensures STAT3 Activation and Tumor Aggressiveness in Glioblastoma

**DOI:** 10.1371/journal.pone.0078728

**Published:** 2013-11-11

**Authors:** Braden C. McFarland, Suk W. Hong, Rajani Rajbhandari, George B. Twitty, G. Kenneth Gray, Hao Yu, Etty N. Benveniste, Susan E. Nozell

**Affiliations:** Department of Cell, Developmental and Integrative Biology, University of Alabama at Birmingham, Birmingham, Alabama, United States of America; University of Michigan School of Medicine, United States of America

## Abstract

Glioblastoma (GBM) is the most aggressive, neurologically destructive and deadly tumor of the central nervous system (CNS). In GBM, the transcription factors NF-κB and STAT3 are aberrantly activated and associated with tumor cell proliferation, survival, invasion and chemoresistance. In addition, common activators of NF-κB and STAT3, including TNF-α and IL-6, respectively, are abundantly expressed in GBM tumors. Herein, we sought to elucidate the signaling crosstalk that occurs between the NF-κB and STAT3 pathways in GBM tumors. Using cultured GBM cell lines as well as primary human GBM xenografts, we elucidated the signaling crosstalk between the NF-κB and STAT3 pathways utilizing approaches that either a) reduce NF-κB p65 expression, b) inhibit NF-κB activation, c) interfere with IL-6 signaling, or d) inhibit STAT3 activation. Using the clinically relevant human GBM xenograft model, we assessed the efficacy of inhibiting NF-κB and/or STAT3 alone or in combination in mice bearing intracranial xenograft tumors *in vivo*. We demonstrate that TNF-α-induced activation of NF-κB is sufficient to induce IL-6 expression, activate STAT3, and elevate STAT3 target gene expression in GBM cell lines and human GBM xenografts *in vitro*. Moreover, the combined inhibition of NF-κB and STAT3 signaling significantly increases survival of mice bearing intracranial tumors. We propose that in GBM, the activation of NF-κB ensures subsequent STAT3 activation through the expression of IL-6. These data verify that pharmacological interventions to effectively inhibit the activity of both NF-κB and STAT3 transcription factors must be used in order to reduce glioma size and aggressiveness.

## Introduction

Gliomas are the most commonly occurring type of malignant primary brain tumor within the central nervous system (CNS). The World Health Organization (WHO) classifies astrocytomas into four grades based on histological features with grade III (anaplastic astrocytoma) and grade IV (primary and secondary glioblastoma (GBM)) both being malignant gliomas [1]. These tumors are characterized by their highly invasive and neurologically destructive nature, as well as their ability to diffusively infiltrate normal brain tissue [2]. To date, GBMs remain incurable and are nearly one hundred percent fatal [3]. Based on recent efforts to characterize genetic abnormalities in these tumors, GBMs are organized into four molecular subtypes: proneural, neural, classical and mesenchymal (Mes) [4]. With respect to the Mes subtype of GBM, STAT3 has been identified as a master regulator of these tumors [5], [6], and Mes tumors also display enhanced NF-κB activity and inflammation-associated genes [4]. In contrast, epidermal growth factor receptor (EGFR) amplifications and/or mutations are common events in the classical subtype [4], and studies have shown that STAT3 can be alternatively activated via EGFR signaling [7]. Therefore, it is apparent that both NF-κB and STAT3 are key players in the pathology of GBM tumors.

STAT3 is a member of the STAT family of transcription factors [8], [9]. In most cells, STAT3 lies dormant in the cytoplasm. On the cell surface, specific receptors bind IL-6, LIF or other IL-6 family members, and activate receptor-associated JAK1 and JAK2 protein tyrosine kinases on the intracellular face. Once activated, JAKs phosphorylate and activate STAT3, promoting its dimerization and translocation to the nucleus. In the nucleus, STAT3 induces the expression of genes such as SOCS3, VEGF, c-Myc and IL-6 [10]. Moreover, STAT3 activation promotes growth and survival through the expression of anti-apoptotic proteins, such as Bcl-2, Bcl-xL and Mcl-1 [9].

The NF-κB family contains five structurally similar members (p65/RelA, RelB, c-Rel, NF-κB1/p50 and NF-κB/p52) [11], [12]. In the CNS, the predominant form of NF-κB is a dimer composed of p65 and p50, and it is found inactive and sequestered in the cytosol through interactions with the Inhibitor of NF-κB (IκB) proteins. Exposure to a variety of stimuli, including TNF-α, leads to phosphorylation and activation of the kinase complex composed of IKKα, IKKβ and NEMO. This complex, once activated, phosphorylates IκBα, which is then ubiquitinated and degraded by the proteasome. The NF-κB p65 protein is then free to be phosphorylated by IKKα/β, translocate into the nucleus and induce the expression of target genes such as IL-6 and LIF as well as anti-apoptotic genes including Bcl-2 and Bcl-xL. Additionally, NF-κB induces the expression of its negative regulator IκBα [10], [12].

NF-κB and STAT3 are important transcription factors involved in mediating inflammatory and immune responses, and have also been linked to many cancers, including GBM [9], [10], [12]–[16]. Our lab and others have shown that both NF-κB and STAT3 proteins and activity are elevated in GBM patient samples compared to control tissue [17]–[20]. Moreover, it has also been determined that the levels of numerous NF-κB and STAT3 target genes are elevated in GBM [17], [21]–[23]. Furthermore, there is evidence demonstrating a cooperative interaction between NF-κB and STAT3 transcription factors. In other cell types, STAT3 was shown to maintain NF-κB activity by prolonging NF-κB nuclear retention through recruitment of the p300 acetyltransferase and the subsequent acetylation of NF-κB p65 [24]. More recently, it has been demonstrated that STAT3 binds to the transactivation domain (TAD) of NF-κB p65 to cooperatively induce gene expression in glioma cells following radiation [25]. Therefore, the intent of this study was to determine how the NF-κB and STAT3 signaling pathways influence each other in the context of GBM. We demonstrate that TNF-α activated NF-κB can induce and/or maintain STAT3 activation through the expression of IL-6 in GBM. Furthermore, pharmacological inhibition of both NF-κB and STAT3 is necessary in order to prolong survival and decrease tumor burden *in vivo*.

## Materials and Methods

### Ethics Statement

All *in vivo* experiments in mice (female athymic nude, Harlan Laboratories) were performed with the approval of the University of Alabama at Birmingham Institutional Animal Care and Use Committee (APNs #120908862, #120309368 and #120809198). All surgeries were performed under ketamine/xylazine anesthesia and all efforts were made to minimize suffering.

### Reagents

Recombinant human TNF-α, IL-6 and sIL-6R were purchased from R&D Systems (Minneapolis, MN). Antibodies against phospho-p65 Ser536 (p-p65 (S536)), phospho-STAT3 Tyr705 (p-STAT3 (Y705)), STAT3, phospho-IKKα/β (S176/180) and IKKα/β were purchased from Cell Signaling Technology (1∶1,000; Beverly, MA). Antibodies against p65 and SOCS3 were purchased from Santa Cruz Biotechnology (1∶500; Santa Cruz, CA), and antibodies against GAPDH were purchased from Abcam (1∶10,000; Cambridge, MA). Neutralizing antibodies against human gp130 and polyclonal goat IgG were purchased from R&D Systems (Minneapolis, MN). AZD1480, a JAK1/2 inhibitor, was synthesized and generously provided by AstraZeneca [26]. Withaferin A (WA), an NF-κB inhibitor, was purchased from Enzo Life Sciences (Farmingdale, NY). Doxycycline (Dox) food was purchased from Bio-Serve (Frenchtown, NJ).

### Cell Lines and Human GBM Xenografts

The U251-MG and U87-MG human glioma cell lines were obtained from ATCC and maintained as previously described [18]. Murine glioma cell line (GL261) was a generous gift from Dr. Yancey Gillespie, University of Alabama at Birmingham, which was originally purchased from the NCI-Frederick Cancer Research National Tumor Repository as previously described [27]. U251-TR/*sh-p65* cells that stably express the Tet repressor protein and inducibly express shRNA specific for p65 (*sh-p65*) were generated as previously described [18]. Human GBM xenograft flank tumors propagated in athymic nude mice were maintained by the UAB Brain Tumor Core Facility (Dr. Yancey Gillespie). The human GBM xenograft tumors (xenolines) maintained by the core facility are patient derived tumors that are immediately propagated in the flanks of nude mice, rather than in cell culture. This method of serially passaging human tumors *in vivo* has been demonstrated as a more clinically relevant model to study GBM [28]. Three xenolines (X1016, X1046 and X1066) were chosen based on high activation of both NF-κB and STAT3 as described previously [29]. Xenograft flank tumors (X1046, X1066 and X1016) were dissociated into single cells for brief cell culture analysis (no more than 3 passages in culture). For disaggregation into single cells, flank tumors were removed, washed with PBS, minced, and disaggregated using an enzyme solution consisting of Trypsin/EDTA (5%), DNase I (0.1%) and Collagenase (1%) in PBS. Cells were passed through a 40 µm filter and plated in Neurobasal media with Amphotericin (1%), B27 Supplement, Gentamycin (0.25%), L-glutamine (260 mM), EGF (10 ng/ml), and FGF (10 ng/ml) and cultured as neurospheres as previously described [29].

### Immunoblotting

Cells were lysed in RIPA buffer with protease and phosphatase inhibitors, and protein concentration was determined using the BioRad Assay. Equivalent amounts of total protein (20–30 µg for all except SOCS3 (100 µg)) were analyzed by SDS-PAGE with antibodies specified above, as previously described [18].

### Total RNA Isolation and Quantitative RT-PCR

Total RNA was isolated as previously described [18]. One µg of total RNA was reverse transcribed and analyzed by quantitative PCR using following primers/probe sets purchased from Applied Biosystems (Foster City, CA): *IL-6* (Hs00174131_m1), *LIF* (Hs00171455_m1), *SOCS3* (Hs02330328_s1), *P65* (Hs00153294_m1), *NFKBIA* (IκBα) (Hs00153283_m1), *BIRC3* (cIAP2) (Hs00985031_g1) and *18S* (Hs99999901_s1). Reactions for each sample were performed in triplicate using a PCR protocol (95°C activation for 10 min followed by 40 cycles of 95°C for 15 s and 60°C for 1 min) in an ABI StepOnePlus Detection System (Applied Biosystems, Foster City, CA). ΔΔCt values for genes examined were determined using Ct values generated by StepOnePlus software (Applied Biosystems).

### ELISA

Supernatants were collected from cells serum starved for 4–16 h before treatment with or without TNF-α, and quantification of secreted IL-6 and LIF was assayed by ELISA (Biolegend, San Diego, CA).

### Cell Viability

Xenograft cells were plated in 96 well plates at a density of 2,000 cells/well. Cells were treated with the indicated doses of AZD1480 and/or WA for 48 h, and the WST-1 cell viability assay was performed as previously described [29].

### Chromatin Immunoprecipitation (ChIP)

ChIP assays were performed as previously described [18], [30]. Immunoprecipitation was performed with 5 µg of the appropriate antibodies, and the immune complexes were absorbed with protein A beads or protein G beads (Upstate Cell Signaling Solutions, Charlottesville, VA) blocked with bovine serum albumin and salmon sperm DNA. After pre-clearing and prior to immunoprecipitation, equal amounts of sonicated DNA (10% volume of each sample) were removed for later analysis by quantitative PCR (Input). Immunoprecipitated DNA was subjected to quantitative PCR using primers specific for *IL-6* and *SOCS3* promoters. The following primers were used: IL-6-Pro-F 5′-GCG ATG GAG TCA GAG GAA AC-3′, IL-6-Pro-R 5′-TGA GGC TAG CGC TAA GAA GC -3′, SOCS3-Pro-F 5′-CCC CCA ACT TCT CAT TCA CA-3′, and SOCS3-Pro-R 5′-GGT CTC CCC TCT GGA ATC TG-3′. Results are presented as percentage of input.

### Subcutaneous Flank Tumors

U251-TR/*sh-p65* cells were injected into the flanks of athymic nude mice as previously described [29]. Briefly, 5×10^6^ U251-TR/*sh-p65* cells in a 1∶4 ratio of matrigel to PBS were injected in the flanks of nude mice. Tumors were measured on the indicated days using digital calipers, and tumor volume was calculated using the following equation: v = (0.5 × longest diameter x shortest diameter^2^). Tumors were allowed to grow until reaching an average of approximately 100–200 mm^3^. At this time, mice were randomized into four groups (**Fig. S1A**) to receive treatment for the duration of the experiment. Treatment groups consisted of vehicle only, food supplemented with doxycycline (Dox), a tetracycline analog to induce *p65* shRNA expression, AZD1480 (50 mg/kg) in methylcellulose via oral gavage once a day, or both Dox food and AZD1480. Mice were euthanized, and tumors excised and snap frozen for analyses of RNA and protein.

### Intracranial Tumors

Xenograft tumors were dissociated into single cells as described above. Xenograft X1016 cells (3×10^5^) in 5 µl methylcellulose were injected intracranially into athymic nude mice, 2 mm anterior and 1 mm lateral to the bregma at a depth of 2 mm over 2 minutes for adequate perfusion as previously described [29]. Intracranial tumors were allowed to establish for 3 days before beginning treatment with vehicle, AZD1480 (30 mg/kg, IP, BID), WA (4 mg/kg, IP, alternate days), or both AZD1480 and WA for 3 weeks. Mice were monitored for survival and euthanized upon moribund.

### Statistical Analysis

ANOVA analysis was done on appropriate multivariable analyses with Student-Newman-Keuls post hoc analysis, students t-test for comparison of 2 conditions, and the LogRank test was used for Kaplan–Meier survival curves. Values represent the mean ± SD unless noted otherwise. p<0.05 was considered statistically significant.

## Results

### TNF-α Activates NF-κB and STAT3 in Glioma Cells

**Figure 1:**
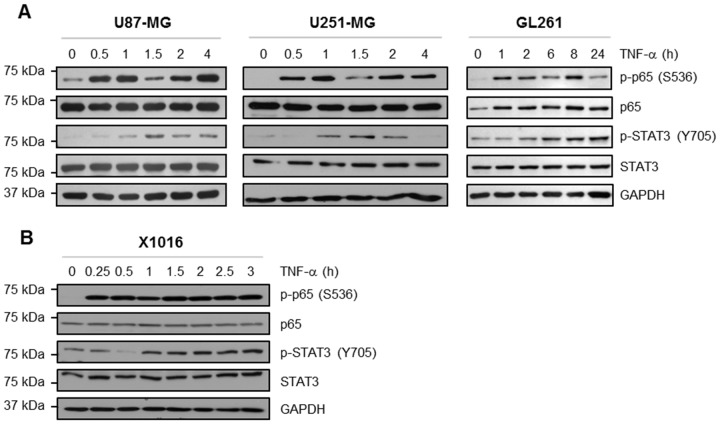
TNF-α Treatment Activates NF-κB and STAT3 in Glioma Cells. **A&B,** U87-MG, U251-MG, GL261 and X1016 cells were treated with TNF-α (10 ng/ml) for the indicated times, lysed and immunoblotted with the indicated Ab.

TNF-α is a pro-inflammatory cytokine and potent activator of NF-κB [11]. As shown in **Fig. 1A**, cultured human (U87-MG and U251-MG) and murine glioma cells (GL261) were treated with TNF-α for various times, and phosphorylation of NF-κB p65 protein was examined. In U87-MG and U251-MG, NF-κB activation was observed within 30 min. A reproducible decrease in the levels of p-p65 occurred at 1.5 h post-TNF-α stimulation, which is consistent with previous reports [31], [32]. In addition, a delayed activation of STAT3 following TNF-α stimulation was observed at 1–2 h, indicating activation of the STAT3 pathway. In GL261 glioma cells, TNF-α-induced NF-κB activation was observed at 1 h, and STAT3 activation at 6 h. STAT3 is typically activated within 5–15 min of stimulation by members of the IL-6 family of cytokines. However, TNF-α is not a classical activator of STAT3. Therefore, the delayed phosphorylation of STAT3 following TNF-α stimulation suggests an indirect, potential feed-forward activation of STAT3.

To confirm that these data are not phenomena of cultured cell lines, we assessed TNF-α-induced STAT3 activation in human GBM cells maintained in an environment similar to the tumor microenvironment. First, we analyzed patient-derived GBM cells that have been serially maintained as xenografts in the flanks of nude mice [29]. Unlike glioma cell lines, GBM samples maintained as xenografts retain the genetic profiles of the tumors from which they were derived [28]. Xenograft 1016 (X1016) tumor was briefly cultured as neurospheres *in vitro* and stimulated with TNF-α for various times. TNF-α induced the rapid and sustained phosphorylation of NF-κB p65 in X1016 cells (**Fig. 1B**). Additionally, the levels of phosphorylated STAT3 were elevated within 1–1.5 h of TNF-α stimulation, and remained elevated until 3 h. Collectively, these data indicate that TNF-α-induced STAT3 activation is conserved in cells derived from primary GBM tumors.

### TNF-α Induces IL-6 and LIF Expression in Glioma Cells

**Figure 2:**
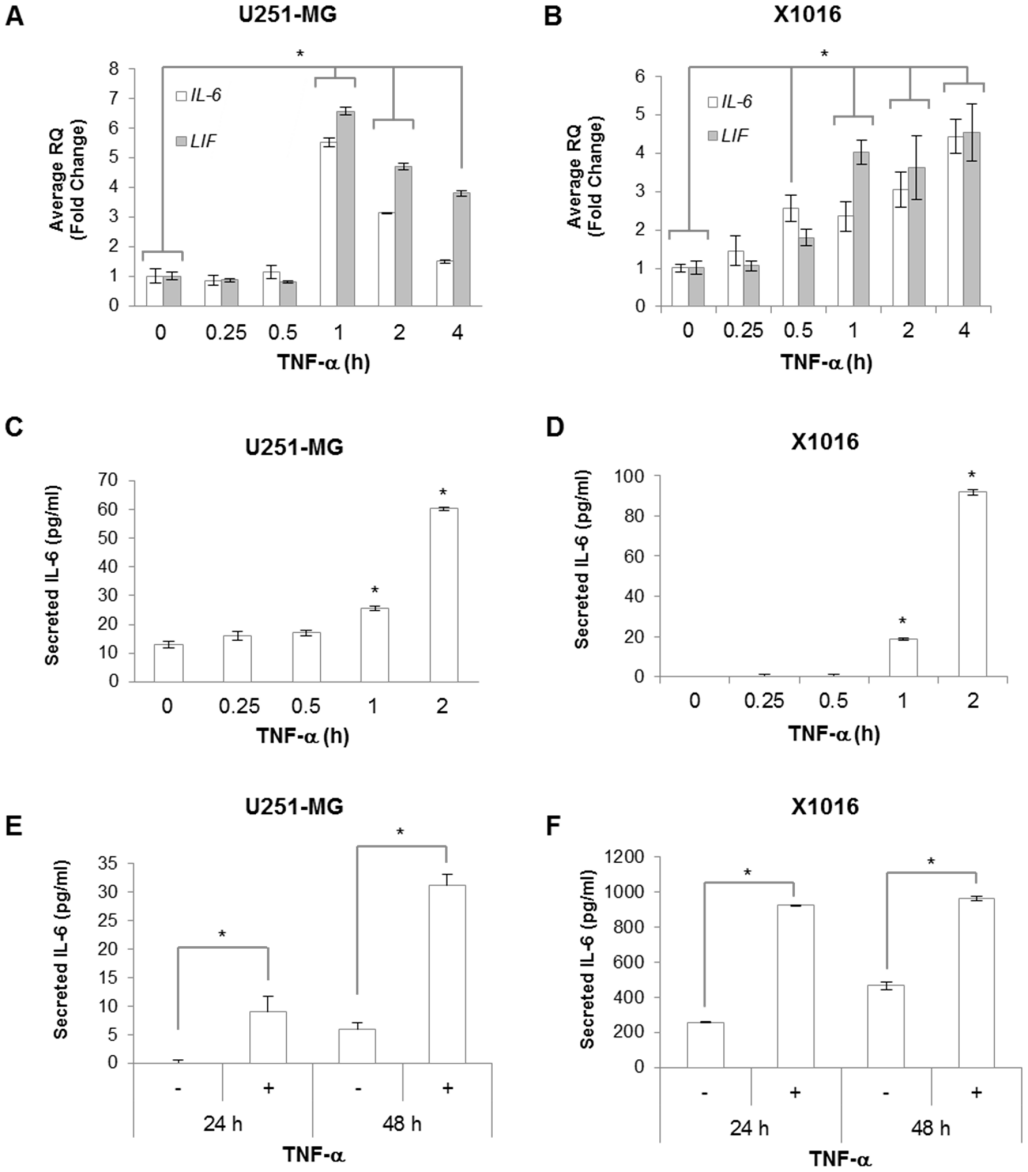
TNF-α Induces IL-6 and LIF Expression in Glioma Cells. **A&B,** U251-MG and X1016 cells were treated with TNF-α (10 ng/ml) for the indicated times. RNA was isolated followed by generation of cDNA, and qRT-PCR was performed for the indicated genes. Data are shown as replicates of three and the experiment repeated with similar results observed. *, p<0.05. **C-F,** Supernatants were collected from U251-MG and X1016 cells stimulated with TNF-α for the indicated times, and quantitation of secreted IL-6 was measured by ELISA. Data are shown as replicates of three and the experiment repeated with similar results observed. *, p<0.05.

Because IL-6 and LIF are the best characterized activators of STAT3 [33], and it is known that TNF-α can induce IL-6 expression, this suggests that TNF-α induction of IL-6 and/or LIF production might be one mechanism by which STAT3 becomes activated. To test this, U251-MG and X1016 cells were treated with TNF-α for various times and *IL-6* and *LIF* mRNA levels evaluated by quantitative RT-PCR (qRT-PCR). *IL-6* and *LIF* mRNA levels were significantly increased by 1 h of TNF-α stimulation and remained elevated out to 4 h (**Figs. 2A & B**). Next, cells were treated with TNF-α for various times and supernatants collected to measure secreted IL-6 and LIF. In the absence of any stimulation, U251-MG and X1016 cells express low levels of IL-6, and in response to TNF-α, IL-6 levels were significantly increased by 1–2 h (**Figs. 2C & D**). These levels continued to rise and remained elevated at 48 h post-stimulation (**Figs. 2E & F**). Relative amounts of IL-6 secreted depend on experimental conditions, whereas cell density is equivalent only within individual experiments, thus yielding different levels of IL-6 in the untreated conditions between experiments. Although similar analyses were performed to evaluate LIF expression, the levels of LIF protein fell below the detectable range within the time period assessed. Because of the low LIF protein levels, we focused our studies on IL-6. These data indicate that TNF-α induces the expression and secretion of IL-6, which may potentially lead to the subsequent activation of STAT3 in glioma cells.

### TNF-α Activates STAT3 and Induces Expression of STAT3 Target Genes

**Figure 3:**
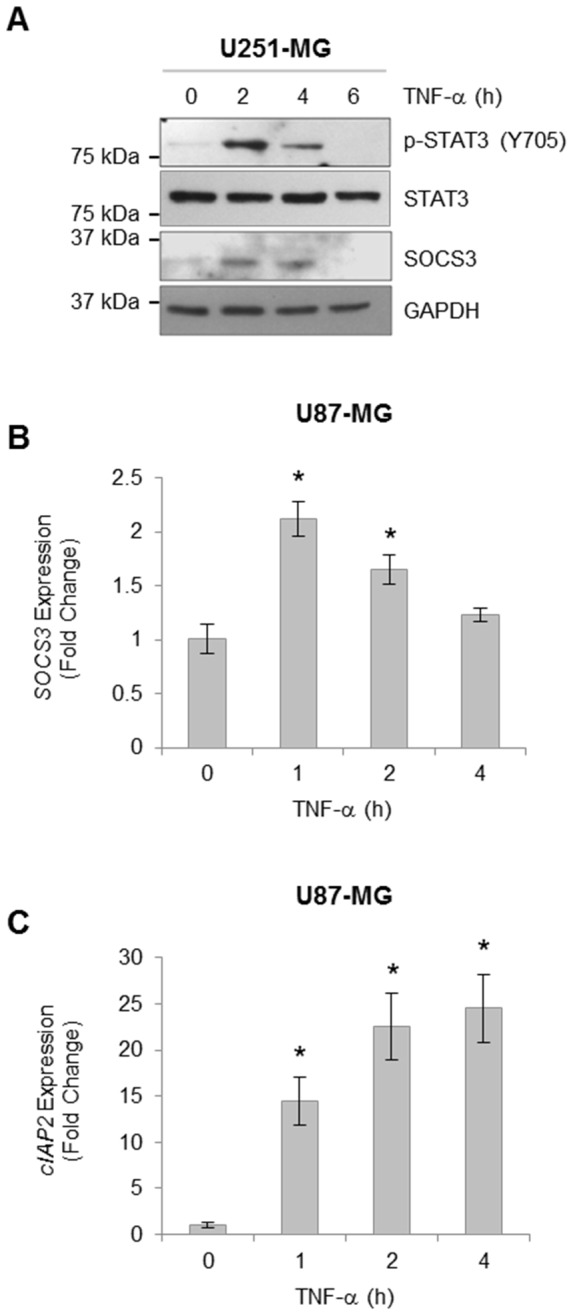
Activation of STAT3 by TNF-α Treatment Induces *SOCS3* and *cIAP2* Expression. **A**, U251-MG cells were stimulated with TNF-α (10 ng/ml) for the indicated times, lysed and immunoblotted with the indicated Ab. **B & C**, U87-MG cells were stimulated with TNF-α (10 ng/ml) for the indicated times. RNA was isolated, followed by generation of cDNA, and qRT-PCR was performed for the indicated genes. Data are shown as replicates of three and the experiment repeated with similar results observed. *, p<0.05.

Next, we evaluated whether TNF-α-induced STAT3 activation coincided with the expression of STAT3 target genes, first examining *SOCS3*. U251-MG cells were treated with TNF-α for various times, and STAT3 activation and SOCS3 levels were evaluated. In response to TNF-α, STAT3 phosphorylation was observed as well as increased levels of SOCS3 protein expression within 2–4 h (**Fig. 3A**). Similar results were obtained by qRT-PCR, demonstrating TNF-α induced *SOCS3* mRNA levels (**Fig. 3B**). In addition to *SOCS3*, an increase in the expression of *cIAP2*, a known STAT3 gene, was also elevated following TNF-α treatment (**Fig. 3C**). Together, these data indicate that TNF-α activation of STAT3 coincides with increased expression of STAT3 target genes.

### IL−6 Activates STAT3 in Glioma Cells

Above, we demonstrated that U251-MG cells expressed IL-6 and exhibited activated STAT3 in response to TNF-α stimulation. Next, we confirmed that this cytokine can directly activate STAT3 in these cells. U251-MG cells were stimulated with IL-6 and soluble IL-6 receptor (sIL-6R) (**Fig. S2A**) for various times. The IL-6R exists as both a membrane-bound and a soluble form (sIL-6R) [34], and the addition of sIL-6R with IL-6 induces optimal JAK/STAT3 activation [35]. STAT3 activation was observed within 15 min of IL-6/sIL-6R stimulation. To confirm that STAT3 activation correlated with changes in target gene expression, U251-MG cells were treated with IL-6/sIL-6R, and total RNA was evaluated for the levels of *SOCS3* mRNA. In response to IL-6/sIL-6R, the levels of *SOCS3* mRNA were increased within 30 min and persisted until 2 h (**Fig. S2B**), confirming functional activation of STAT3.

### TNF-α Promotes Early Recruitment of NF-κB p65 and Late Recruitment of STAT3 to Promoters

**Figure 4:**
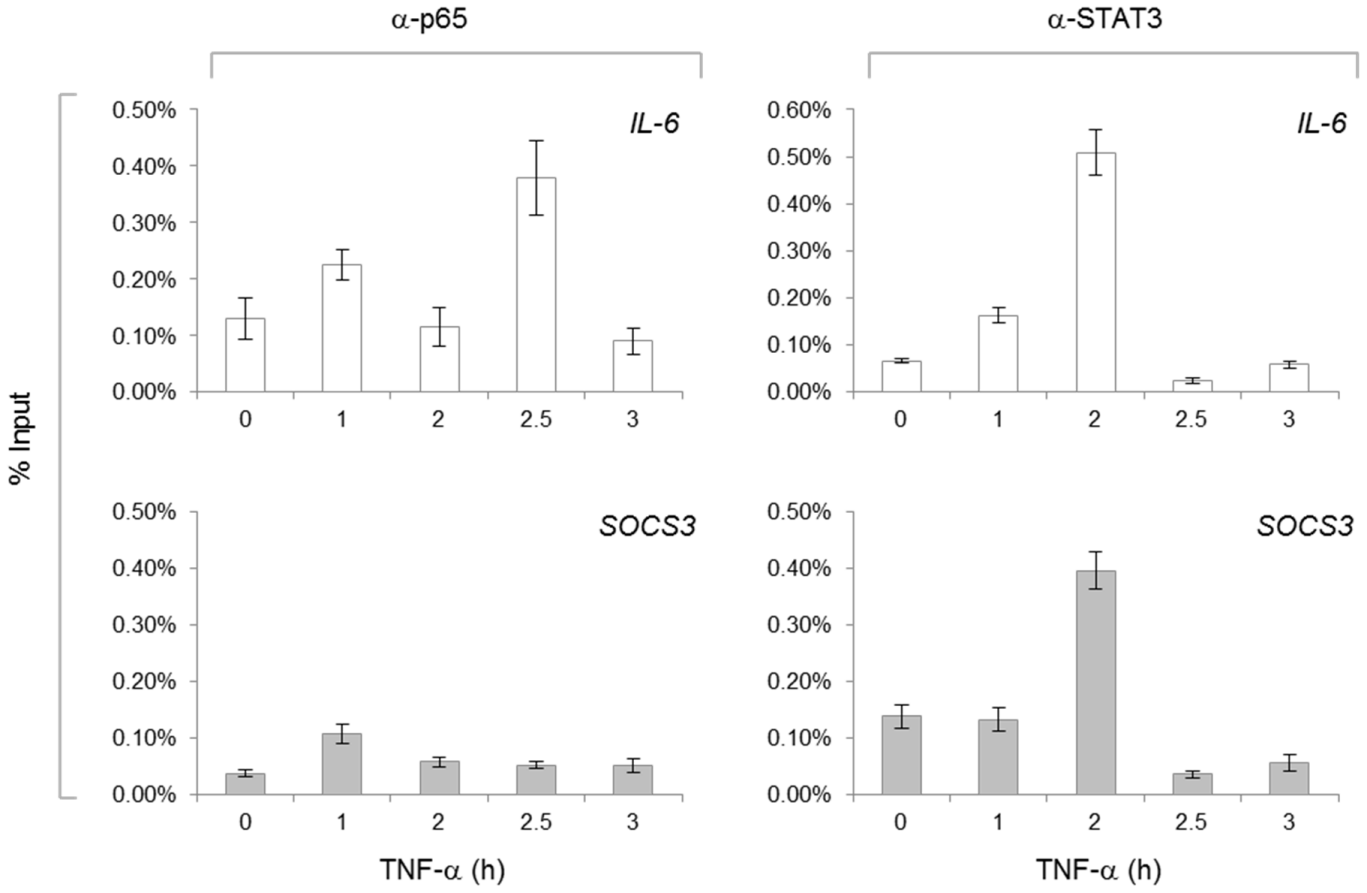
TNF-α Recruits p65 to the *IL-6* Promoter and STAT3 to the *IL-6* and *SOCS3* Promoters. U251-MG cells were grown in the absence or presence of TNF-α (10 ng/ml) for various times, sonicated and soluble chromatin was immunoprecipitated with antibodies specific for p65 or STAT3. Immunoprecipitated DNA was then analyzed by qRT-PCR using primers specific for the *IL-6* and *SOCS3* promoters. Each sample was normalized to genomic DNA isolated from cells that were cross-linked and processed, yet did not incur the immunoprecipitation step. The results are shown as percentages of input, replicates of three, and error bars represent standard deviation.

Thus far, our hypotheses and data are premised on the idea that TNF-α activated NF-κB is primarily responsible for IL-6 induction early (within 1 h), and that IL-6 activated STAT3 is primarily responsible for STAT3 target gene expression later (2 h). To confirm these events and their times of occurrence, ChIP assays were performed. U251-MG cells were grown in the absence or presence of TNF-α for various times, and sonicated, soluble chromatin was immunoprecipitated with antibodies specific for p65 or STAT3. Immunoprecipitated DNA was then analyzed by qRT-PCR using primers specific for the *IL-6* and *SOCS3* promoters. As shown in **Fig. 4**, modest levels of p65 are detected at the two promoters in the absence of TNF-α stimulation (0 h). Upon TNF-α stimulation, there were increased levels of p65 at the *IL-6* promoter at 1 h and again at 2.5 h post-TNF-α stimulation. There is a slight increase in p65 at the *SOCS3* promoter at 1 h. However, this minor increase does not correspond to the time at which SOCS3 mRNA and protein expression occurs, suggesting p65 alone is insufficient for SOCS3 expression. Modest levels of STAT3 are detected at the *IL-6* and *SOCS3* promoters in the absence of TNF-α stimulation, and these levels remain relatively unaffected until 2 h post-TNF-α stimulation. At 2 h post-TNF-α, the levels of STAT3 at the *IL-6* and *SOCS3* promoters are substantially increased, and this coincides with elevated *IL-6* and *SOCS3* mRNA expression. We and others have previously shown that STAT3, in addition to NF-κB, can also drive the expression of IL-6 [10], which is supported by the recruitment of STAT3 to the *IL-6* promoter at 2 h. Overall, this suggests that in response to immediate TNF-α stimulation, NF-κB is activated and induces the expression of *IL-6*, which activates STAT3 and then induces a new wave of *IL-6* and *SOCS3* expression.

### TNF-α-induced STAT3 Activation and Gene Expression are Dependent on NF-κB p65

**Figure 5:**
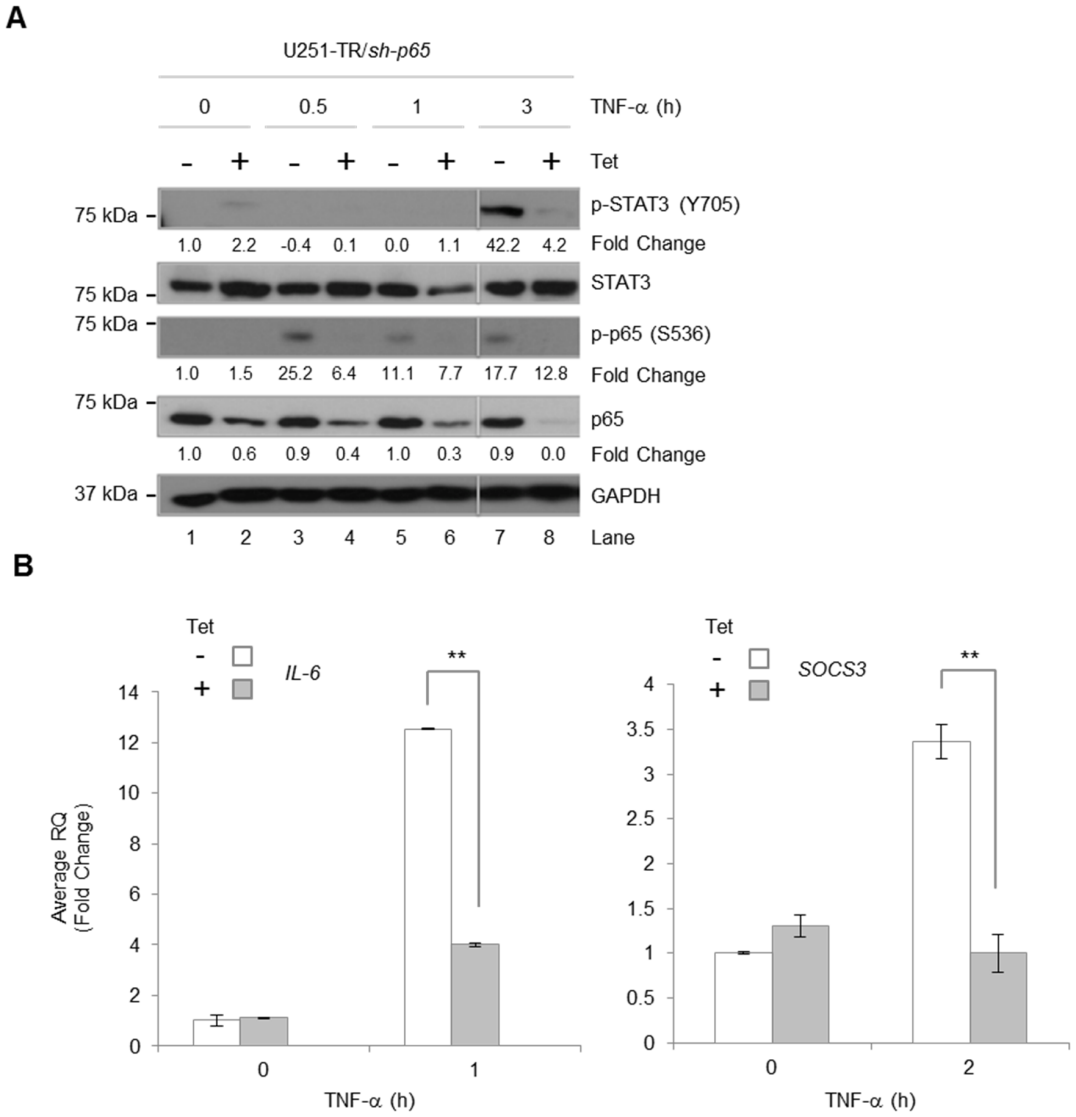
TNF-α-induced STAT3 Activation is Dependent on NF-κB p65. **A & B**, U251-TR/*sh-p65* cells were incubated with tetracycline (Tet) for 48 h prior to stimulation with TNF-α (10 ng/ml) for the indicated times. Cells were lysed and immunoblotted with the indicated Ab (A) or RNA was isolated, followed by generation of cDNA, and qRT-PCR was performed for the indicated genes (B). Densitometric values of p-STAT3, p-p65 and total p65 were normalized to total STAT3, total p65 and GAPDH, respectively. Data are shown as replicates of three. **, p<0.01.

Next, we addressed whether TNF-α-induced STAT3 requires activation of NF-κB. Previously, we generated U251-TR/*sh-p65* cells that express shRNA molecules specific for *p65* when grown in the presence of tetracycline (Tet) [18], [21]. U251-TR/*sh-p65* cells were grown in the absence or presence of Tet for 48 h to reduce endogenous p65 levels (**Fig. 5A**, **lanes 2, 4, 6 and 8**). TNF-α-induced phosphorylation of p65 was inhibited in cells expressing *p65* specific shRNA (**Fig. 5A**, **lanes 4, 6 and 8**). In response to TNF-α, STAT3 was phosphorylated at 3 h which was inhibited by reduced p65 expression (**Fig. 5A**, **lane 8**). Together, these data indicate that NF-κB p65 is required for TNF-α-induced STAT3 phosphorylation.

To assess whether TNF-α-induced downstream IL-6 expression requires NF-κB p65 protein, U251-TR/*sh-p65* cells were grown in the absence or presence of Tet for 48 h, and then stimulated with TNF-α for the indicated times (**Fig. 5B**). After TNF-α stimulation for 1 h, *IL-6* mRNA levels were increased and this enhancement was significantly reduced by diminished NF-κB p65 expression. Moreover, TNF-α-induced *SOCS3* expression at 2 h was also significantly inhibited by the loss of NF-κB p65 (**Fig. 5B**). These data confirm that NF-κB p65 is required for TNF-α-induced STAT3 activation and gene expression of IL-6 and subsequent expression of *SOCS3*.

### TNF-α-induced STAT3 Activation Requires Signaling through the JAK/gp130 Complex

**Figure 6:**
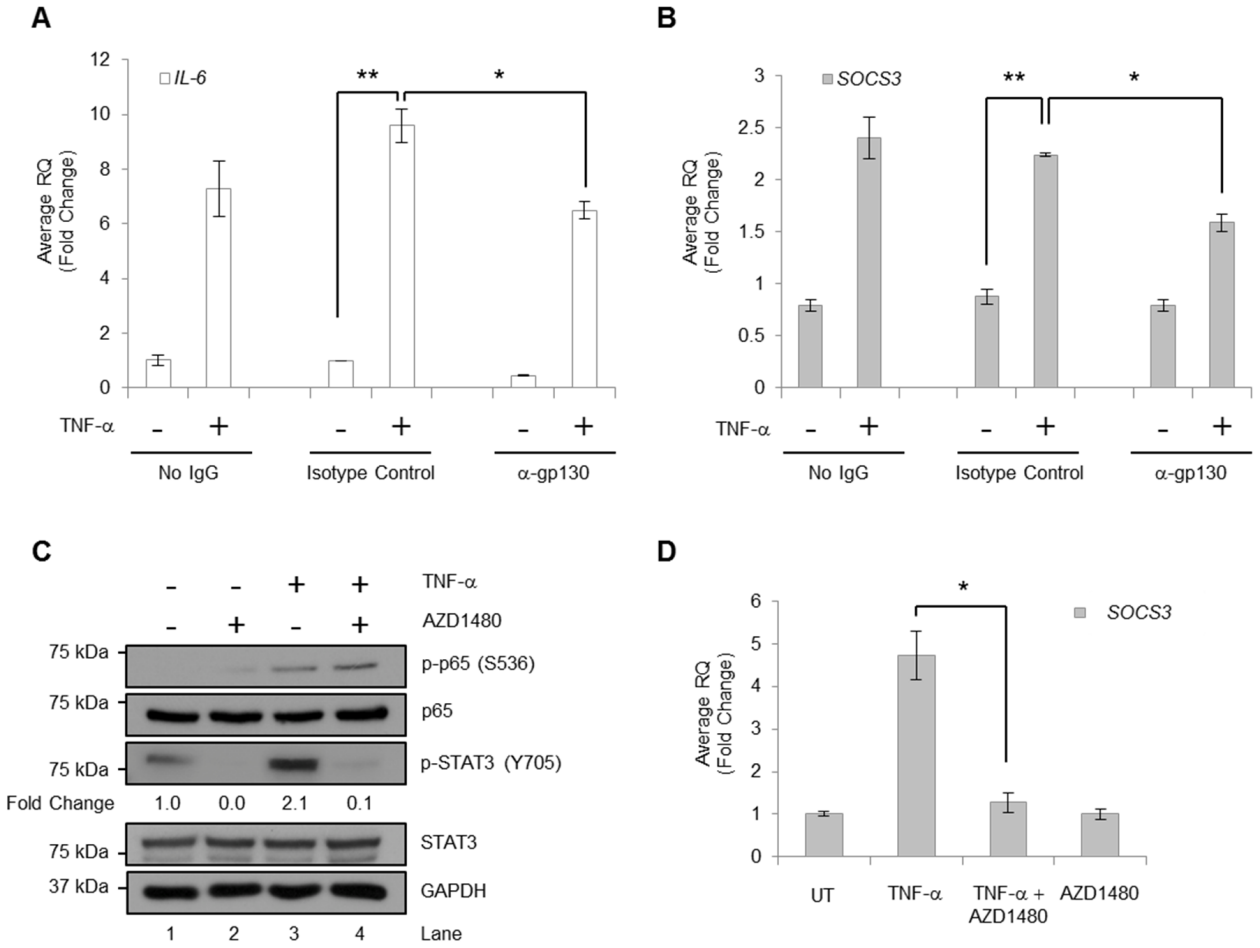
TNF-α-induced STAT3 Activation Requires gp130 and JAK Kinases. **A & B,**U251-MG cells were treated with no IgG, isotype control (1 µg/ml) or anti-gp130 (1 µg/ml) for 2 h followed by treatment with TNF-α (10 ng/ml) for 1 h. RNA was isolated followed by generation of cDNA, and qRT-PCR was performed for the indicated genes. Data are shown as replicates of three. *, p<0.05; **, p<0.01. **C & D**, U251-MG cells were incubated with AZD1480 (1 µM) for 2 h prior to treatment with TNF-α (10 ng/ml) for 2 h. Cells were lysed and immunoblotted with the indicated Ab (C) or RNA was isolated, followed by generation of cDNA, and qRT-PCR performed for *SOCS3* (D). Densitometric values of p-STAT3 were normalized to total STAT3. Data are shown as replicates of three. *, p<0.05.

STAT3 is classically activated when cytokines bind to a receptor complex composed of a ligand-specific receptor and the signal transducing subunit gp130 [8]. Therefore, we wanted to assess whether TNF-α-induced STAT3 activation also required signaling through this complex. U251-MG cells were grown in the absence or presence of no antibodies (No IgG), isotype control or gp130-specific neutralizing antibodies (α-gp130), and left unstimulated or stimulated with TNF-α. TNF-α induced *IL-6* expression in the absence of antibodies or in the presence of control or gp130-specific antibodies compared to untreated conditions (**Fig. 6A**). These data indicate that TNF-α-induced *IL-6* expression is not mediated by gp130 and is largely dependent on NF-κB activation by TNF-α. However, TNF-α-induced *SOCS3* expression was significantly reduced by the presence of gp130-specific antibodies (**Fig. 6B**), indicating that TNF-α-induced *SOCS3* expression is at least partially mediated by gp130, further suggesting that IL-6 activated STAT3 signals through gp130 and is responsible for the majority of *SOCS3* expression.

To evaluate the requirement for JAK activity in TNF-α-induced STAT3 phosphorylation, we utilized a pharmacological inhibitor of JAK1/2 kinases, AZD1480 [29], [36]. U251-MG cells were pre-treated with AZD1480 prior to stimulation with TNF-α. As expected, TNF-α induced the phosphorylation of NF-κB p65, which is unaffected by AZD1480 (**Fig. 6C** **, lane 4**). However, both basal and TNF-α enhanced STAT3 phosphorylation is inhibited by AZD1480, suggesting a requirement for JAK1/2 (**Fig. 6C** **, lanes 2 and 4**). To demonstrate that these conditions correlate with changes in gene expression, we evaluated *SOCS3* mRNA levels by qRT-PCR at 2 h post-TNF-α stimulation. In U251-MG cells, levels of *SOCS3* are enhanced by TNF-α stimulation and significantly reduced by pre-treatment with AZD1480 (**Fig. 6D**). This suggests that JAK1/2 kinases are required for TNF-α-induced STAT3 activation, and subsequent *SOCS3* gene expression.

### Loss of Global STAT3 Activation but not Tumor-Specific NF-κB Activation Impairs Tumor Growth

**Figure 7:**
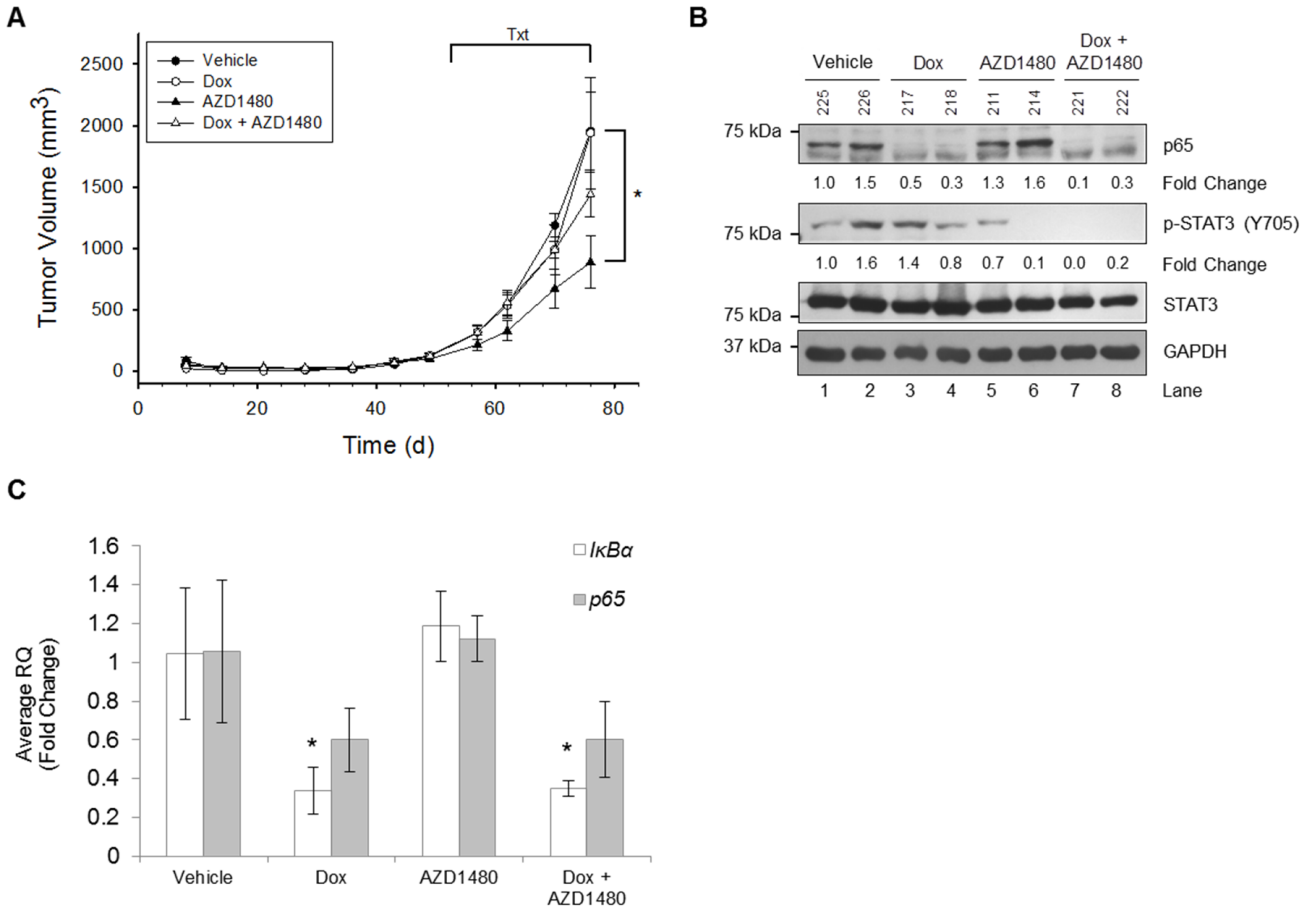
Loss of Global STAT3 Activation but not Tumor-Specific NF-κB Activation Impairs Tumor Growth. **A,** U251-TR/*sh-p65* cells were injected subcutaneously into nude mice, and tumor growth was measured on the indicated days. At day 53, mice were randomized into 4 groups (n = 4 per group) to begin treatment (Txt) regimens. Treatment groups consisted of vehicle only (vehicle), food supplemented with doxycycline (Dox, a tetracycline analog to induce p65 shRNA expression), AZD1480 (50 mg/kg) in methylcellulose via oral gavage once a day, or both Dox food and AZD1480 (Dox+AZD1480). Data represent mean ± SEM (*, p<0.05, Students t-test at day 76). **B,** Frozen tumor samples were homogenized and immunoblotted with the indicated Ab. Representative tumor samples from each group are shown. Densitometric values of total p65 and p-STAT3 were normalized to GAPDH and total STAT3, respectively. **C,** Frozen tumor samples were homogenized, and RNA isolated, followed by generation of cDNA, and qRT-PCR performed for the indicated genes (n = 2 tumors per condition, replicates of three per tumor). *, p<0.05.

Our data thus far indicate that once activated, NF-κB has the potential to promote STAT3 activation. Moreover, several studies have demonstrated that inhibition of STAT3 activity reduces glioma growth [29], [37]–[39]. Therefore, we sought to assess whether local inhibition of NF-κB within tumor cells only, global inhibition of STAT3 activity in all cells, or both were sufficient to reduce tumor growth *in vivo*. U251-TR/*sh-p65* cells were injected into the flanks of nude mice, and the experimental design is outlined in **Fig. S1A**. TNF-α was not used to activate NF-κB, rather, we relied on the pro-inflammatory tumor microenvironment to trigger the initial NF-κB activation. To reduce endogenous NF-κB p65 levels in tumor cells only, mice were provided with food containing doxycycline, a tetracycline derivative. To globally inhibit STAT3 activation, mice received AZD1480 once a day via oral gavage. Finally, to inhibit both tumor cell NF-κB and global STAT3, mice received the combination of Dox supplemented food and AZD1480 treatment. Subcutaneous tumor volume was measured on the indicated days. Mice receiving no treatment (Vehicle) or Dox food only demonstrated the largest tumor volumes and were indistinguishable from one another (**Fig. 7A**). In contrast, mice receiving AZD1480 alone exhibited the smallest tumor volumes, consistent with our previous findings that STAT3 inhibition reduced glioma tumor volume [29]. Those mice receiving Dox and AZD1480 exhibited tumor volumes slightly, but not significantly, larger than those found in mice receiving AZD1480 alone.

To demonstrate that inhibition of STAT3 and NF-κB activation was achieved *in vivo*, subcutaneous tumors were resected and analyzed by immunoblotting and qRT-PCR. As shown in **Fig. 7B**, tumors from mice receiving Dox demonstrated reduced levels of NF-κB p65 protein (**Fig. 7B** **, lanes 3–4 and 7–8**) as well as reduced levels of both *p65* and *IκBα* mRNA (**Fig. 7C**). Furthermore, tumors from mice receiving AZD1480, alone or in combination with Dox food, exhibited reduced levels of STAT3 phosphorylation compared to vehicle (**Fig. 7B** **, lanes 5–8**). These data suggest that the loss of intratumoral NF-κB is insufficient to inhibit global STAT3 activation in the tumor, nor is it able to enhance the reductions in tumor volume already mediated by STAT3 inhibition via AZD1480.

### Pharmacological Inhibition Disrupts NF-κB and STAT3 Signaling in GBM Xenograft Cells In Vitro

**Figure 8:**
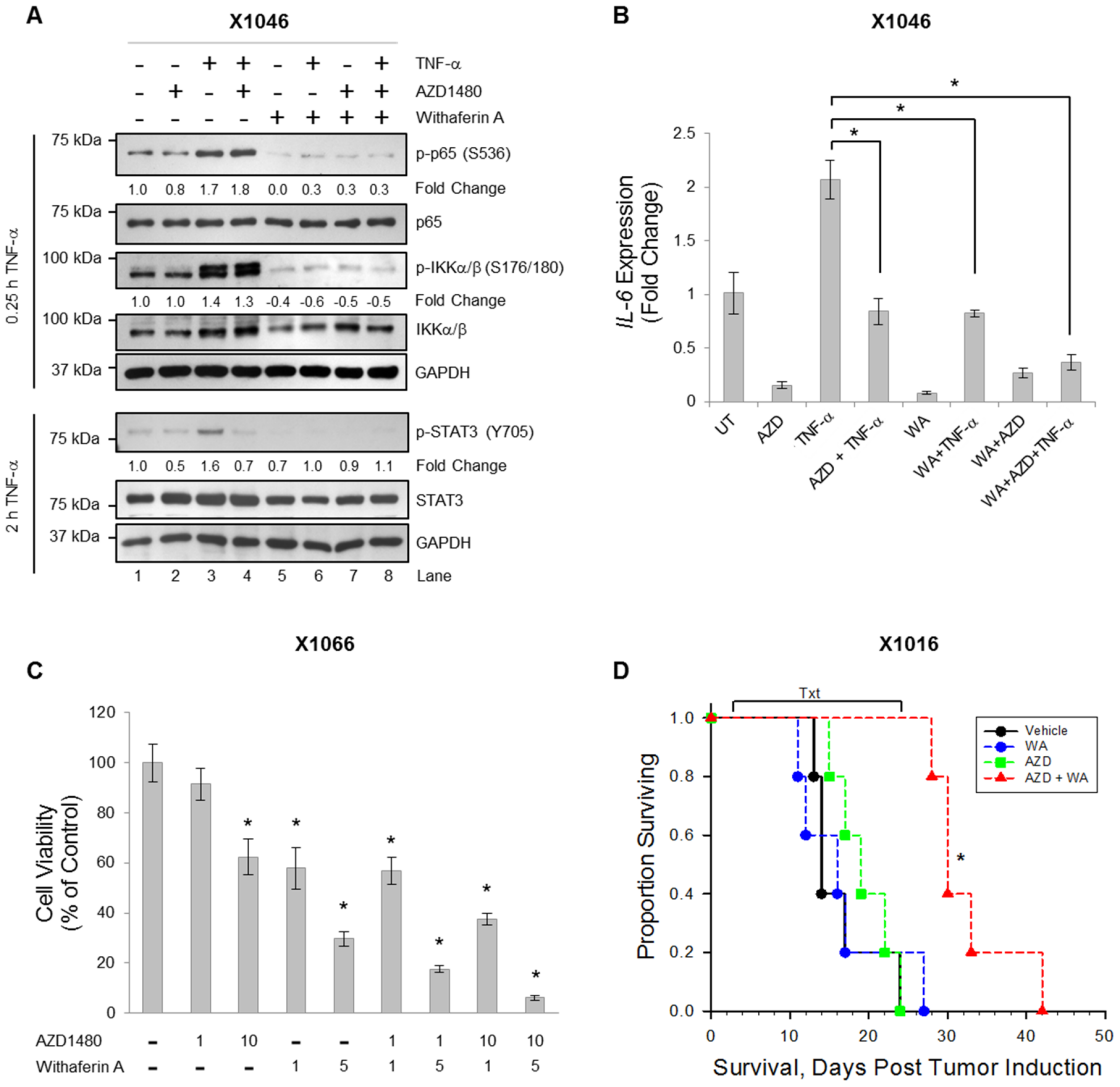
Combined Pharmacological Inhibition Disrupts NF-κB and STAT3 Signaling *In Vitro* and Prolongs Survival *In Vivo*. **A & B,** Xenograft X1046 cells were disaggregated into single cells and briefly propagated as neurospheres *in vitro*. Cells were pre-treated with AZD1480 (1 µM) and/or WA (5 µM) for 2 h prior to TNF-α (10 ng/ml) for 0.25 or 2 h (A) or 2 h (B). Cells were lysed and immunoblotted with the indicated Ab (A), or RNA was isolated followed by generation of cDNA and qRT-PCR was performed for *IL-6* (B). Densitometric values of p-p65, p-IKKα/β and p-STAT3 were normalized to total p65, total IKKα/β and total STAT3, respectively. Data are shown as replicates of three. *, p<0.05. **C,** Xenograft X1066 cells were treated with the indicated doses of AZD1480 and/or WA for 48 h, and the WST-1 cell viability assay was performed. Data are shown as replicates of three. *p<0.05. **D,** Nude mice were injected intracranially with Xenograft X1016 cells. Starting at day 3, mice were treated with vehicle (n = 5), AZD1480 (30 mg/kg, twice a day, n = 5), WA (4 mg/kg, alternate days, n = 5) or both AZD+WA (n = 5) for three weeks. Survival was measured, and mice were euthanized at moribund. *, p<0.05 (LogRank).

From the data presented above, we hypothesize that within the tumor microenvironment, NF-κB in other cell types is likely activated and thus contributes to STAT3 activation and/or tumor growth in that capacity. To test this hypothesis, we used pharmacological inhibitors to suppress global activation of NF-κB, STAT3, or both, in the intracranial GBM model using human GBM xenografts. We have previously shown that AZD1480 is an effective inhibitor of STAT3 signaling in xenograft cells both *in vitro* and *in vivo* [29]. In order to inhibit NF-κB signaling we used Withaferin A (WA), a known inhibitor of NF-κB signaling, that can readily cross the blood-brain barrier and has demonstrated anti-tumor effects in glioma [40]–[44]. Specifically, WA disrupts the NEMO/IKKβ complex interaction [41], which is essential for canonical NF-κB pathway activation, and therefore prevents phosphorylation of IKKα/β as well as NF-κB p65. In Xenograft X1046 cells, TNF-α induced the phosphorylation of the upstream kinase, IKKα/β, and NF-κB p65 within 15 min (**Fig. 8A** **, lane 3**), and these events were blocked by pre-treatment with WA *in vitro* (**Fig. 8A** **, lane 6**). Similarly, TNF-α induced the phosphorylation of STAT3 at 2 h (**Fig. 8A** **, lane 3**), which was inhibited by pre-treatment with AZD1480 (**lane 4**) or WA (**lane 6**). We verified that downstream gene transcription was also inhibited using xenograft cells treated with TNF-α for 2 h. *IL-6* expression was evaluated by qRT-PCR and significantly reduced in the presence of WA, AZD1480 or both *in vitro* (**Fig. 8B**). To assess functional effects, we evaluated viability of xenograft cells following inhibition of NF-κB and STAT3 activation. Xenograft X1066 cells were treated with the indicated doses of AZD1480 and/or WA for 48 h. Treatment with AZD1480 (10 µM) or WA (1 & 5 µM) alone statistically inhibited viability **(** **Fig. 8C** **)**. However, combination AZD1480 and WA treatment exerted an additive inhibition of viability in these cells. Overall, these data confirm that AZD1480 and WA effectively inhibit NF-κB and STAT3 signaling and decrease viability of xenograft cells *in vitro*.

### Inhibition of Global NF-κB and STAT3 Activity Increases Survival in an Orthotopic Model of Glioma

To evaluate the effect of pharmacological inhibition of NF-κB and/or STAT3 *in vivo*, we proceeded to the orthotopic intracranial xenograft model of glioma. Since both STAT3 and NF-κB aberrations have been linked to GBM, we previously verified that the xenografts exhibit constitutive NF-κB and STAT3 activation [29] (data not shown). To evaluate whether pharmacological inhibition of both NF-κB and STAT3 activation prolongs survival of mice bearing intracranial tumors, xenograft cells (X1016) were disaggregated from the flanks of nude mice, dissociated into single cells and injected in the brains of nude mice. Beginning on day 3, mice received treatments consisting of vehicle control, AZD1480, WA, or both AZD1480 and WA. The experimental design is described in **Fig. S1B**. Mice receiving single agent AZD1480 or WA alone displayed survival curves not significantly different from vehicle control (**Fig. 8D**). However, mice receiving both AZD1480 and WA displayed prolonged survival that was statistically significant compared to vehicle control (**Fig. 8D**). These data indicate that in order to effectively disrupt STAT3 and NF-κB pathways *in vivo*, combination treatment must be employed.

## Discussion

**Figure 9:**
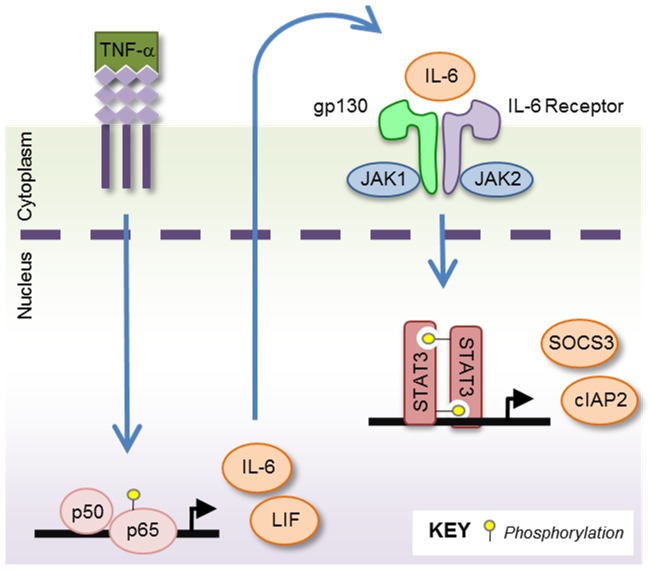
Signaling Schematic Illustrating the Cycle of Cooperation Between NF-κB and STAT3. NF-κB and STAT3 are competent to ensure activation of themselves and each other, either in an autocrine and/or paracrine manner. Upon stimulation with TNF-α, the NF-κB pathway becomes activated, as shown by the phosphorylation and nuclear translocation of NF-κB p65 and transcription of NF-κB genes, including IL-6 and LIF. Newly synthesized IL-6 is secreted by the cells, and binds in an autocrine or paracrine manner to the IL-6 receptor. This leads to activation of the IL-6R/gp130 complex and the intracellular kinases JAK1/2. STAT3 proteins then become phosphorylated by JAK1/2, dimerize, enter the nucleus and begin the transcription of STAT3 driven genes such as SOCS3 and cIAP2.

In this report, we have explored the signaling crosstalk that occurs between the NF-κB and STAT3 pathways in GBM tumors, which is illustrated in **Fig. 9**. In cultured glioma cell lines as well as human GBM xenografts, we found that TNF-α treatment activated both the NF-κB and STAT3 pathways, in a temporal manner. This was observed by an early activation of NF-κB followed by delayed activation of STAT3, indicating a potential autocrine or feed-forward mechanism. In GBM, TNF-α induced expression of IL-6, which we propose then signals in an autocrine and/or paracrine manner to activate STAT3. Therefore, in a pro-inflammatory environment such as GBM, activation of NF-κB feeds forward to ensure activation of STAT3. Once activated, STAT3 induces the expression of various genes, including IL-6, continuing the vicious crosstalk. We demonstrate that TNF-α-induced activation of STAT3 requires NF-κB p65. Utilizing cells that inducibly decrease NF-κB p65 protein, we found that TNF-α-induced STAT3 activation and downstream gene transcription is diminished when NF-κB p65 levels are reduced. Furthermore, we examined the role of the gp130/cytokine receptor complex in TNF-α-induced STAT3 activation. We found that GBM cells treated with antibodies blocking the gp130 subunit or pharmacological inhibition of JAK kinases were unable to activate STAT3 following TNF-α stimulation. Our *in vitro* data suggest that by inhibiting NF-κB signaling, and thus the expression of IL-6, subsequent STAT3 activation will be inhibited.

The phenomenon of signaling crosstalk between NF-κB and STAT3 has previously been suggested by others; however, it has never been definitely elucidated. For example, Tanabe et al. [45] observed that TNF-α stimulation led to phosphorylation of STAT3 in rat glioma cells; however, the investigators did not evaluate the possible feed-forward mechanisms and concluded that TNF-α activated NF-κB, STAT3 and p38 MAPK, resulting in the production of IL-6. Similarly, in diffuse B-cell lymphoma, constitutive NF-κB activation induced the production of IL-6, leading to a feed-forward mechanism activating STAT3 [46]. Thus, these reports, like ours, indicate that NF-κB activation of STAT3 may be conserved across multiple cancers, including GBM. Moreover, our study is the first report to demonstrate that activation of NF-κB leads to the feed-forward activation of STAT3, and to test the efficacy of combined inhibition of NF-κB and STAT3 *in vivo* in GBM.

*In vivo*, examination of the crosstalk signaling between NF-κB and STAT3 proved more complex. In subcutaneous flank tumors of U251-TR/*sh-p65* cells, we observed that loss of NF-κB p65 in tumor cells only was insufficient to inhibit tumor growth. Furthermore, the loss of intratumoral NF-κB p65 did not further enhance inhibition of global STAT3 activation, or reduce tumor growth as mediated by AZD1480, which we observed previously [29]. This indicates other mechanisms may activate STAT3, including other cell types within the tumor microenvironment. In fact, a probable source of NF-κB activation within tumors is through TNF-α stimulation. TNF-α is present in glioma tumors and is thought to be mainly secreted by endogenous microglia or tumor-associated macrophages [47], [48]. Studies have suggested that following STAT3 inhibition, the levels of TNF-α increase, promoting a more inflammatory macrophage/microglia resulting in inhibition of tumor growth [49], [50]. However, these cells, although intended to help promote tumor clearing, can inadvertently aid in the promotion and expansion of tumor growth through secreted cytokines including TNF-α and IL-6. Therefore, targeting a broader NF-κB population, for example in microglia/macrophages, is an important therapeutic avenue to consider.

Next, we examined the more clinically relevant xenograft model to investigate the role of NF-κB and STAT3 *in vivo*. We proceeded to determine if combined inhibition of STAT3 and NF-κB was necessary to prolong survival in mice bearing intracranial tumors. We exploited an inhibitor of NF-κB signaling, WA, to globally inhibit NF-κB within tumors and the microenvironment. In a previous report, it was demonstrated that treatment with WA alone (12 mg/kg) prolonged survival in mice bearing intracranial xenograft tumors [43]. In our experiments, we utilized a lower dosage of WA (4 mg/kg) and therefore did not see an anti-tumor effect of WA treatment alone. Previously, we demonstrated that AZD1480 alone (50 mg/kg) increases survival of mice with xenograft intracranial tumors [29]. In the current study, we utilized a lower dosage (30 mg/kg) and did not see prolonged survival with AZD1480 alone. Due to the lowered dosage of AZD1480 and WA, we did not see a significantly enhanced survival with single agent therapy. We anticipate that if higher doses of AZD1480 and WA alone were administered, an anti-tumor effect would be observed. However, using lower doses of single agents in combination allows us to observe potential additive effects in prolonging survival of mice, while reducing possible toxicities due to high dose of single therapy approaches. Indeed, our combinatorial approached revealed prolonged survival of mice bearing intracranial tumors when both drugs were used simultaneously, even at these lower doses. Furthermore, we confirmed that NF-κB and STAT3 activation are inhibited in intracranial tumors by these drugs (data not shown). Interestingly, further analysis revealed that the xenograft tumor we tested *in vivo* (X1016) also has amplification of EGFR (unpublished data from G.Y.G, UAB Brain Tumor Core Facility) in addition to high levels of activated STAT3 and NF-κB, suggesting a probable classical genetic subtype. Therefore, we have confirmed that combination inhibition of NF-κB and STAT3 is therapeutically beneficial to this subtype of tumors. Whether there is a preferential benefit among Mes specific tumors has yet to be determined. In fact, a recent report utilized a multi-sampling technique and determined that most patient tumors display different GBM subtypes within the same tumor [51]. Therefore, regardless of the genetic subtype of GBM tumors, targeting NF-κB and STAT3 is an important avenue to consider.

In conclusion, we have closely examined the signaling crosstalk that occurs between the NF-κB and STAT3 pathways in GBM. We found that activation of NF-κB signaling leads to the expression of IL-6, which ultimately leads to the activation of STAT3. *In vitro,* inhibition of NF-κB signaling will inhibit and prevent the downstream activation of STAT3. However, *in vivo*, both NF-κB and STAT3 appear to be activated in response to multiple stimuli in the tumor microenvironment, and single agent therapies prove less effective. To our knowledge, this is the first report to test pharmacological inhibitors in combination to target the crosstalk signaling between NF-κB and STAT3 in GBM. Our data further validate NF-κB and STAT3 as important therapeutic targets for patients with GBM.

## Supporting Information

Figure S1 **Experimental** **Design of** ***In Vivo*** **Subcutaneous Flank and Intracranial Experiments.** **A**, U251-TR/*sh-p65* cells were injected into the flanks of nude mice. Mice were randomized into 4 groups. Group 1 received vehicle only. Group 2 received Dox food to decrease NF-κB p65 in tumor cells. Group 3 received AZD1480 treatment to inhibit global STAT3 activation. Group 4 received both Dox food and AZD1480 treatment. **B**, Human GBM xenograft cells were injected intracranially into nude mice. Mice were randomized into 4 groups. Group 1 received vehicle only. Group 2 received Withaferin A (WA) treatment to inhibit global NF-κB activation. Group 3 received AZD1480 treatment to inhibit global STAT3 activation. Group 4 received both WA and AZD1480 treatment.
(TIF)

Figure S2 **IL-6 Activates STAT3 in Glioma Cells. A**, U251-MG cells were incubated with IL-6 (10 ng/ml)/sIL-6R (25 ng/ml) for the indicated times. Cells were lysed and immunoblotted with the indicated Ab. **B**, U251-MG cells were incubated with IL-6 (10 ng/ml)/sIL-6R (25 ng/ml) for the indicated times. RNA was isolated, followed by generation of cDNA and qRT-PCR was performed for *SOCS3*. *, p<0.05.
(TIF)
