# Supplementary figures and images for: NF-κB-Induced IL-6 Ensures STAT3 Activation and Tumor Aggressiveness in Glioblastoma
Source: PLoS One. 2013 Nov 11;8(11):e78728. doi: 10.1371/journal.pone.0078728 (PMC3823708; doi:10.1371/journal.pone.0078728)

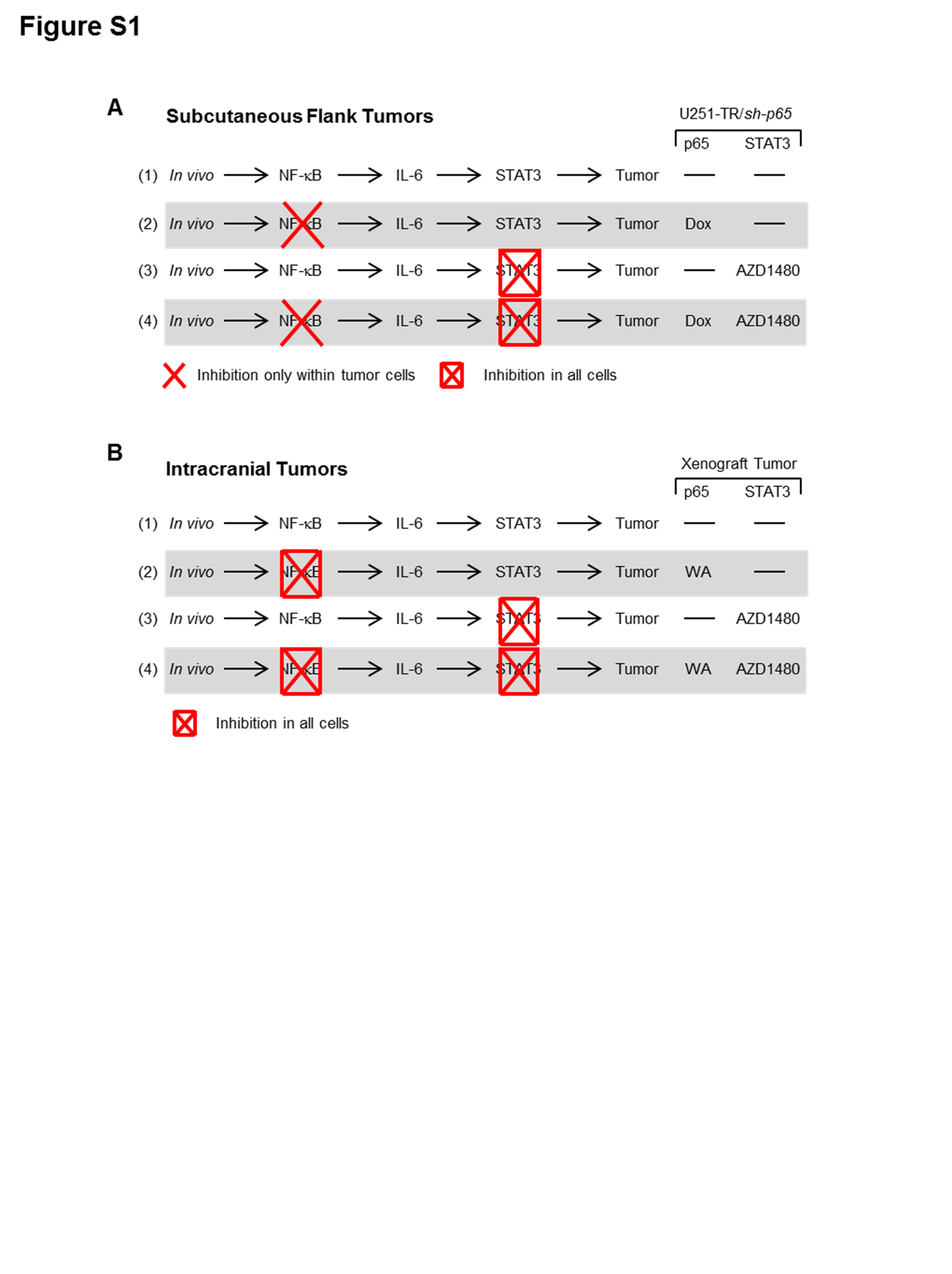

Supplement: Figure S1 — Experimental Design of In Vivo Subcutaneous Flank and Intracranial Experiments. A, U251-TR/sh-p65 cells were injected into the flanks of nude mice. Mice were randomized into 4 groups. Group 1 received vehicle only. Group 2 received Dox food to decrease NF-κB p65 in tumor cells. Group 3 received AZD1480 treatment to inhibit global STAT3 activation. Group 4 received both Dox food and AZD1480 treatment. B, Human GBM xenograft cells were injected intracranially into nude mice. Mice were randomized into 4 groups. Group 1 received vehicle only. Group 2 received Withaferin A (WA) treatment to inhibit global NF-κB activation. Group 3 received AZD1480 treatment to inhibit global STAT3 activation. Group 4 received both WA and AZD1480 treatment. (TIF) [file pone.0078728.s001.tif]

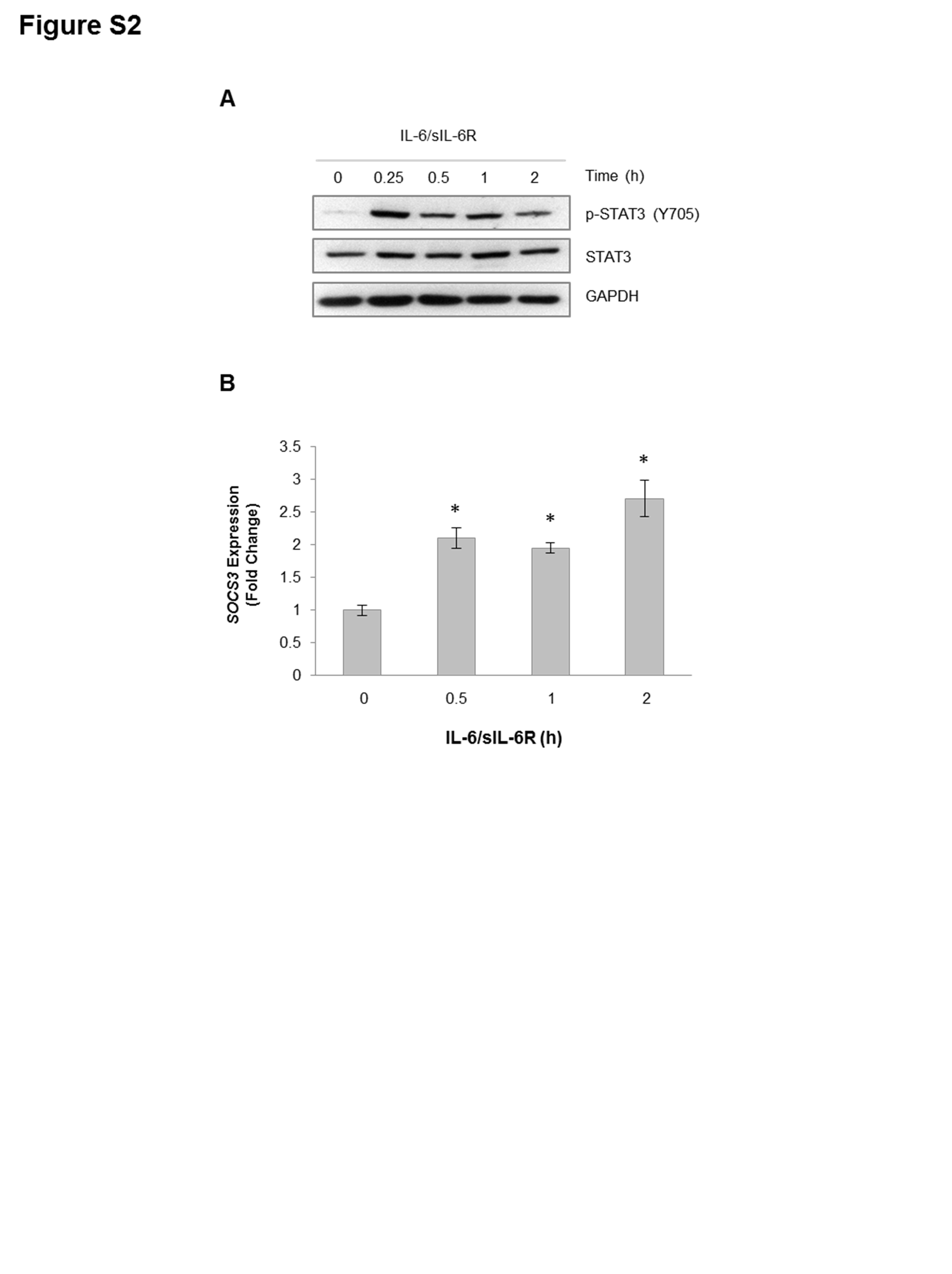

Supplement: Figure S2 — IL-6 Activates STAT3 in Glioma Cells. A, U251-MG cells were incubated with IL-6 (10 ng/ml)/sIL-6R (25 ng/ml) for the indicated times. Cells were lysed and immunoblotted with the indicated Ab. B, U251-MG cells were incubated with IL-6 (10 ng/ml)/sIL-6R (25 ng/ml) for the indicated times. RNA was isolated, followed by generation of cDNA and qRT-PCR was performed for SOCS3. *, p<0.05. (TIF) [file pone.0078728.s002.tif]
